# A Review of Fatigue Crack Growth for Pipeline Steels Exposed to Hydrogen

**DOI:** 10.6028/jres.115.030

**Published:** 2010-12-01

**Authors:** N. Nanninga, A. Slifka, Y. Levy, C. White

**Affiliations:** Materials Reliability Division, National Institute of Standards and Technology, Boulder, CO 80305; Materials Science and Engineering Department, Michigan Technology University, Houghton, MI 49931

**Keywords:** fatigue crack growth, hydrogen, pipelines, steel, review

## Abstract

Hydrogen pipeline systems offer an economical means of storing and transporting energy in the form of hydrogen gas. Pipelines can be used to transport hydrogen that has been generated at solar and wind farms to and from salt cavern storage locations. In addition, pipeline transportation systems will be essential before widespread hydrogen fuel cell vehicle technology becomes a reality. Since hydrogen pipeline use is expected to grow, the mechanical integrity of these pipelines will need to be validated under the presence of pressurized hydrogen. This paper focuses on a review of the fatigue crack growth response of pipeline steels when exposed to gaseous hydrogen environments. Because of defect-tolerant design principles in pipeline structures, it is essential that designers consider hydrogen-assisted fatigue crack growth behavior in these applications.

## 1. Introduction

The ever-increasing monetary and environmental costs of using natural gas and petroleum fuels has led to serious consideration of alternative energy sources. Increases in wind and solar energy appear to be most promising for relieving some of the current energy demands. Large wind farms are beginning to appear in fields across the plains states, while solar farms are becoming more prevalent in the southwestern United States. A critical issue involved with these types of renewable energy sources is that peaks and troughs in energy production occur due to variations in wind and solar cycles that do not necessarily coincide with peaks and troughs in energy demand. In order to alleviate this problem, energy storage capabilities must be in place to balance the production and demand. Gaseous hydrogen offers an efficient way of storing the energy generated by wind and solar farms through networks of pipelines and caverns across the country [1–3]. The energy generated by wind turbines and solar collectors can easily be used to separate water, and the hydrogen can be collected for future use in fuel cells for generating electricity during troughs in wind and solar cycles. In addition to using hydrogen as a storage medium, the application of onboard hydrogen fuel cells and hydrogen internal combustion engines in vehicles is expected to increase [4]. This will also create a demand for hydrogen fuel sources where hydrogen will have to be transported efficiently to end users.

Pipelines offer the most efficient way to transport bulk quantities of gaseous fuel, either from points of production to storage locations or from storage locations to distributed points of end use. It is therefore expected that extensive use of hydrogen pipelines will be needed for both transportation and storage of hydrogen fuel as alternative energy use becomes more prevalent. Unfortunately, the existing network of (mostly natural gas) pipelines is constructed mainly of ferrous materials that are often embrittled by atomic hydrogen. Embrittlement by hydrogen can manifest itself in the form of reduced ductility and notch strengths or subcritical crack growth under monotonic loading, which is called “hydrogen embrittlement” (HE), and increased fatigue crack growth (rate(s)) (FCG(R)). The focus of this paper will be on the latter, namely the effects of atomic hydrogen on the process of fatigue crack growth, which we will call “hydrogen-assisted fatigue crack growth (rate(s))” (HA-FCG(R)). Pipelines and other structural materials are often designed by use of defect-tolerant principles, where knowledge of defect size and FCGR can be used to determine the remaining life of a component. To date, there is only limited information on the effects of hydrogen on FCG in low carbon pipeline steels. Furthermore, the results that do exist suggest that low-strength pipeline alloys are highly susceptible to HA-FCG [5–10].

Current pipeline materials in the U.S. are often regulated according to the American Petroleum Institute’s (API) standard 5L. The main design considerations outlined in API-5L are based on alloy chemistry and tensile strength. Pipeline steel grades are designated by their yield strength (σ*_y_*) in ksi (1 ksi = 6.9 MPa), with X80 indicating an 80 ksi yield strength, etc. The chemical compositions of these steels are fairly simple, with maximum limits on C, Mn, S, P and dispersoid-forming elements such as niobium and vanadium. The variations in strength (e.g., between X42 and X70) do not result primarily from variations in alloy composition, but from variations in the processing route of the steel. Thermo-mechanical processing allows the yield strengths of pipe steels to be tailored through combinations of grain refinement, precipitation hardening (micro-alloying) and phase transformations. The prospect of widespread use of hydrogen pipelines has prompted the American Society of Mechanical Engineers (ASME) to form a committee to investigate and develop a standard specifically for gaseous hydrogen pipelines, ASME B31.12, in addition to a code on hydrogen pressure vessels, ASME Article KD-10 in Division 3 of Sec. VIII. Since defect-tolerant design principles are typically used in pipeline and pressure vessel systems, specifications on FCG are sure to be incorporated in these new codes.

The intent of this paper is to review the existing literature on gaseous hydrogen effects on FCG in low carbon pipeline steels. Most of this research has focused on the X42 grade steel, with some preliminary studies on X70 steel, which was a modern high-strength steel at the time of the studies. Since the 1970’s and 1980’s, when most of the existing literature was published, pipeline steels have evolved from the basic micro-alloyed X70 type to higher strength micro-alloyed steels such as X100 and X120, which have mixed phase microstructures with fine grain or lath sizes. Because these new pipeline materials of higher strength are being considered for widespread use, much of the available data on lower strength pipeline alloys (< X70) is outdated. In fact, there appears to be a serious gap in research on the effects of hydrogen on pipeline steels from the mid 1980’s to the present.

While there is some literature on fatigue crack growth in pipeline steels exposed to electrochemically generated hydrogen in aqueous solutions, this paper will only briefly discuss such results. However, hydrogen embrittlement should follow Sievert’s law and will be influenced by the concentration of atomic hydrogen absorbed in the metal. Once hydrogen has been absorbed into the steel at the crack tip, the mechanism(s) responsible for material damage resulting from electrochemical and gaseous charging will be similar, with most of the experimentally observed differences resulting from differences in the thermodynamics and kinetics of the dissociation reactions influencing the activity of the atomic hydrogen in the crack tip process zone.

## 2. Background

Discussion of the mechanism(s) for HA-FCG in pipeline steels is complicated by the fact that neither the mechanism for fatigue crack growth, nor the mechanism for hydrogen embrittlement (HE) under monotonic loading in these materials, is completely understood. Fatigue crack growth in the absence of explicit environmental influences has been reviewed extensively and will be described only briefly here [11–13]. Furthermore, our discussion will be primarily restricted to what is commonly called “Stage II” growth, which tends to be transgranular in the absence of environmental effects and follows a path normal to the maximum principal tensile stress.

### 2.1 Fatigue Crack Growth in Inert Environments

**Fig. 1:**
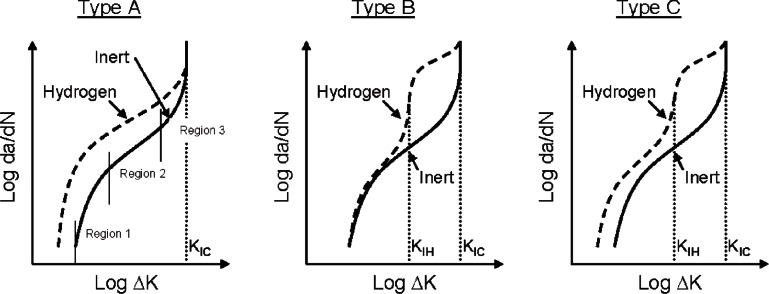
Possible effects of hydrogen on fatigue cracking behavior (from [14, 15])

Stage II fatigue crack growth behavior of structural metals is generally characterized by three distinct regions (see vertical lines in Fig. 1a) [14, 15]. The cyclic stress intensity range (Δ*K*) is defined as the maximum stress intensity per cycle (*K*_max_) minus the minimum stress intensity per cycle (*K*_min_). In Region 1, Δ*K* is so low that Stage II fatigue crack growth is insignificant. Above a threshold stress intensity range (Δ*K*_th_), Stage II cracks begin to exhibit significant growth and the crack growth behavior transitions to Region-2-type growth. Crack growth rates in Region 2 are typically related by the power law function [16]:

(1) $$\frac{\mathrm{da}}{\mathrm{dN}}=A{\left(\Delta K\right)}^{m},$$

where *da*/*dN* is the incremental crack extension per cycle, *A* and *m* are constants, and Δ*K* is the cyclic stress intensity range. The FCGR in Region 2 is governed mainly by crack tip stress intensity levels, but can be affected by testing variables such as stress ratio (*R* = *K*_min_/*K*_max_) [14]. As a fatigue crack grows, *K*_max_ increases to the point where it is essentially equivalent to the critical stress intensity for unstable crack growth (*K_C_*), or *K_IC_* if the crack is propagating under plane strain conditions. Fatigue crack growth in Region 3 occurs as *K_IC_* is approached, and is characterized by significant increases in growth rates.

**Fig. 2:**
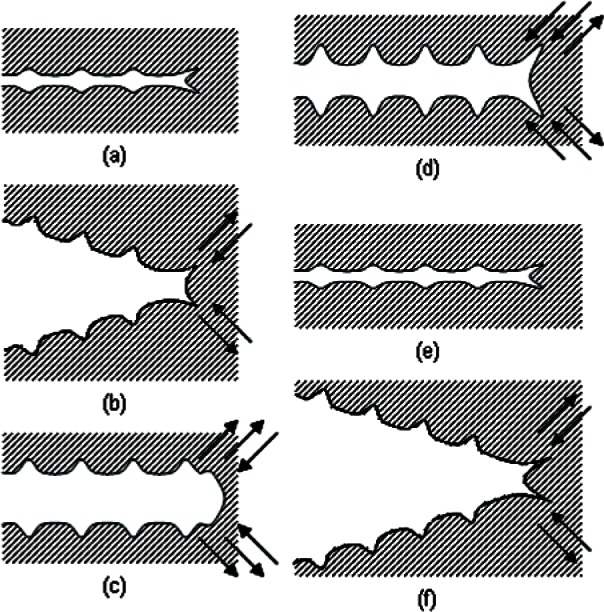
Schematic illustration of Laird’s proposed mechanism for fatigue crack advance through local plasticity at the crack tip (from [11]).

Based on work by Forsyth and Ryder [17] that demonstrated a one-to-one correlation between load cycles, striations on a fatigue fracture surface, and crack advance, both Laird [11] and Pelloux [12] have proposed fatigue crack propagation mechanisms based on details of plastic flow at the tip of a propagating fatigue crack. Laird’s model involves crack advance through local plastic flow during the crack blunting process, with sharpening and work hardening of the crack tip region during crack closure (Fig. 2). Tomkins showed that an analysis of crack tip microplasticity could be used to predict fatigue life [13].

**Fig. 3:**
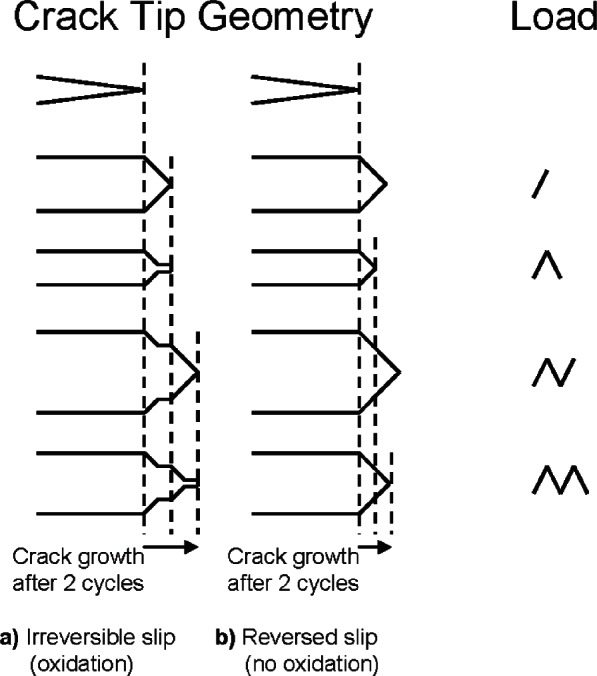
Schematic of Pelloux’s proposed mechanism for fatigue crack propagation through irreversible localized crystallographic slip (a). Pelloux’s mechanism offers an explanation for absence or diminution of striations during fatigue in vacuum, during which the slip process is presumably reversible (b). (from [12]).

Pelloux’s model is also based on details of plastic deformation at the tip of a propagating crack, but focuses on the irreversibility of crystallographic slip at the crack tip (Fig. 3). Both mechanisms explain the presence of fatigue striations that mark the cycle-by-cycle advance of the crack front and emphasize the importance of crack tip plasticity to the crack growth mechanism. Fatigue crack growth rate modeling based on striation formation may not be applicable for materials behavior in hydrogen gas systems. Slip and crack closure in pure hydrogen is expected to be more reversible due to the absence of oxygen and surface oxides films (Fig. 3-b) [18]. In addition, crack advance may occur along grain boundaries in hydrogen, which further complicates the situation [7, 18, 19].

### 2.2 Fatigue Crack Growth in Hydrogen Environments

Hydrogen-assisted fatigue crack growth behavior, like other forms of corrosion-fatigue, has been categorized by Wei and Simmons [15]. Deviation from fatigue crack growth behavior of materials exposed to damaging environments can be characterized as one of three types. These three types of HA-FCG are illustrated in Fig. 1. For Type A, the FCGR in Region 2 may be higher in hydrogen environments, compared to the rate in inert or air environments. In addition, the stress intensity required to activate substantial crack growth may be lower, resulting in a decreased Δ*K_th_*. For the purpose of this review, increases that occur in cyclic crack growth behavior due to atomic hydrogen, such as those exhibited by Type A, will be identified as HA-FCG. Under Type A conditions, the monotonic crack growth threshold in hydrogen (*K_IH_*), the stress intensity above which subcritical crack growth will occur through the material in a statically loaded application exposed to absorbed hydrogen, is essentially equivalent to that of inert environments. This implies that material behaving in this manner may be virtually immune to static HE, and that *K_IH_* is essentially equal to *K_IC_*, but dynamic loading in hydrogen lowers the stress intensity range required for cyclic growth. Conditions leading to Type B fatigue failure occur when a material is susceptible to HE, such that *K_IH_* < *K*_max_ < *K_IC_*, but cyclic loading at *K*_max_ < *K_IH_* does not result in HA-FCG. Many materials may exhibit combined effects of HE and HA-FCG at cyclic stress intensities below *K_IC_*. This behavior is identified as Type C in Fig. 1.

One assumption in the previous discussion, as well as in the details of Fig. 1, is that *K_IH_* values under dynamic loading are representative of those observed in statically loaded tests. For high strength materials, this assumption appears valid. However, for lower strength steels (such as pipeline steels), “active” or rising loads may reduce values of *K_IH_* compared to measurements performed under statically loaded conditions [20]. Since rising loads will be present during every fatigue cycle, it is certainly conceivable that HE-induced subcritical crack growth may be partially responsible for reduced fatigue performance of pipeline steels exposed to pressurized gaseous hydrogen. However, due to the limited understanding of this phenomenon, the discussion in this review will be based on the assumption that static *K_IH_* values are representative of the HE behavior (Type B) and HA-FCG is not a superposition of this behavior.

**Fig. 4:**
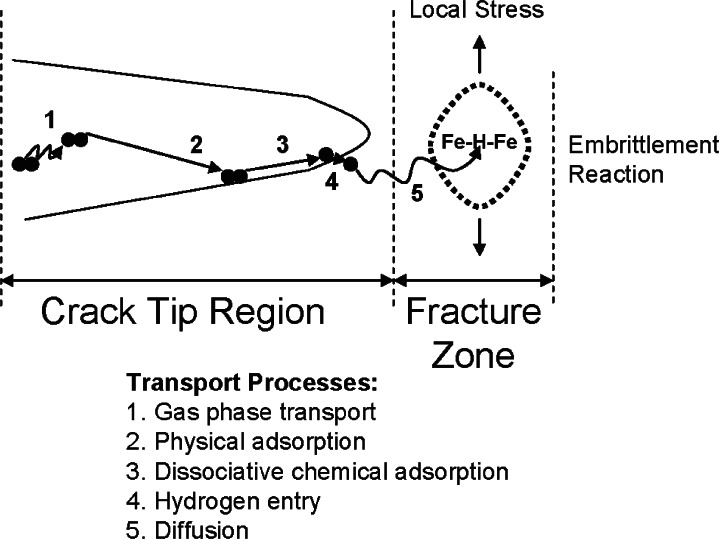
Schematic illustration of processes associated with embrittlement of steels by external hydrogen bearing environments. (From [34]).

Discussion of mechanisms for HA-FCG is complicated by the fact that there is no single accepted mechanism for HE of steel, even during monotonic loading [21–24]. The two most commonly proposed mechanisms relevant to ferritic steels both envision hydrogen enrichment at stressed and/or strained regions such as those ahead of a crack or notch. One of these mechanisms attributes failure in this hydrogen-enriched region to “hydrogen enhanced decohesion” (HEDE) [25–27], and the other attributes it to “hydrogen enhanced local plasticity” (HELP) [28–32]. In both cases, embrittlement by gaseous hydrogen initially requires adsorption of hydrogen gas and formation of atomic hydrogen on the surface of the steel, followed by stress-assisted diffusion to the region of high triaxial stress, such as the region just ahead of a notch or crack tip (see Fig. 4**).** The HELP mechanism can also incorporate hydrogen transport via atmospheres around mobile dislocations. The sequence of events for hydrogen adsorption, absorption and diffusion can be represented as [33]

(2) $$\frac{1}{2}H_{2(g)}\to \frac{1}{2}H_{2(\mathrm{ads})}\to H_{\left(\mathrm{ads}\right)}\to H_{\left(\text{soln}\right)}.$$

Diffusional transport of atomic hydrogen depends on the hydrogen fugacity (function of gas pressure) at the crack tip, the kinetics of the dissociation reaction, and the concentration (activity) and stress field in the steel near the crack tip. The HEDE mechanism envisions a loss in cohesive strength within this hydrogen enriched region as a mechanism for crack advance, and except for the high mobility of atomic hydrogen, has much in common with embrittlement of grain boundaries by segregated impurities. While the mechanics and kinetics of crack propagation in a wide variety of hydrogen rich environments are consistent with the HEDE mechanism, detailed understanding of exactly how the hydrogen enrichment decreases fracture strength is lacking.

The HELP mechanism differs from the HEDE mechanism mainly in the assumed effect of hydrogen enrichment on the properties of the steel. The HELP mechanism assumes that hydrogen enrichment in the vicinity of dislocation cores leads to increased dislocation mobility, and further, that this increased dislocation mobility leads to highly localized failure by plastic instability. Evidence for hydrogen enhanced dislocation mobility in iron and other materials has been provided by *in-situ* transmission electron microscopy studies [21]. Results of some mechanical testing also suggest that hydrogen environments facilitate plastic deformation, but the evidence in these studies is mixed [21, 22, 28]. Unfortunately, a detailed mechanism for crack advance based on hydrogen enhanced plasticity is also lacking.

## 3. HA-FCG Measurements on Pipeline Steels

### 3.1 Baseline HA-FCG of Pipeline Steels

Beyond the uncertainty concerning the mechanism(s) for HE under monotonic loading, our understanding of mechanism(s) for HA-FCG in pipeline steels is further hampered by the relatively sparse literature on this subject. Much of the literature relevant to ferritic pipeline steels derives from research by Holbrook, Cialone and co-workers at Battelle Columbus Laboratories [5–10]. While limited results on X70 pipeline steels, a plain carbon steel of approximately eutectoid composition (AISI 1080), and a relatively pure iron are included in these results, most of the results are for an X42 pipeline steel. This body of work provides clear evidence for enhanced fatigue crack growth rates in hydrogen gas for steels, but there are only a few observations that provide indirect evidence concerning the mechanism for HA-FCG.

**Fig. 5:**
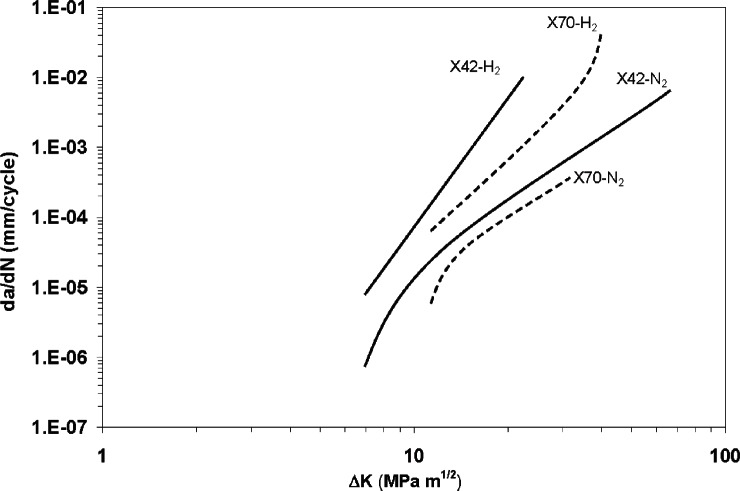
Baseline FCG results in 6.9 MPa hydrogen and nitrogen at *f* = 1 Hz and *R* = 0.1 for X42 (solid lines) and X70 (dashed lines) steels (reproduced using from [9]).

Several key relationships between hydrogen embrittlement, FCG, and FCG testing variables were accounted for in the research conducted by Cialone and Holbrook. Testing variables that may affect FCG in hydrogen include frequency, pressure, stress ratio, alloy microstructure, and strength. Each of these variables will be addressed individually in subsequent sections. Baseline results for fatigue crack growth rates in hydrogen and nitrogen for X42 and X70 alloys at gas pressures of 6.9 MPa (1,000 psi), stress ratio of 0.1 and frequency of 1 Hz are provided in Fig. 5. The investigators reported little dependence of FCG on cyclic frequencies between 0.1 Hz and 10 Hz and therefore performed most of their tests at 1 Hz. The FCGR were determined over stress intensity ranges between 20 MPa m^1/2^ and 70 MPa m^1/2^. The baseline test results for the X42 pipeline steel in hydrogen exhibited FCGR increases of up to 150 times those observed in nitrogen at stress intensity ranges near 20 MPa m^1/2^. At lower stress intensities, the deviation in FCGR between the hydrogen and inert environments decreased, indicating that the threshold stress values may be less affected by hydrogen. FCGR for the X70 alloy were also accelerated in the high pressure gaseous hydrogen environment, but the increases were about half of those exhibited by the X42 alloy.

Cotrill and King have also studied HA-FCG in a C-Mn structural steel by flowing hydrogen gas across the specimen surface and using a frequency of 0.1 Hz and stress ratio of 0.1 [35]. The FCGR at low Δ*K*(≈22 MPa m^1/2^) deviated only slightly between specimens tested in air and hydrogen, but at higher Δ*K*(≈40 MPa m^1/2^) the rate in hydrogen increased by almost twenty times the rate in air. The increase in FCGR at higher Δ*K* values was attributed to static HE at values where *K*_max_ was above *K_IH_*. However, the FCGR was linear throughout the entire stress intensity range and did not show Type B behavior (Fig. 1b), as would be expected if this were the case. The effect of gaseous hydrogen (near 101 Pa) on FCG for a 1020 steel has also been studied at intermediate stress intensity ranges at a frequency of 1 Hz and stress ratio of ≈ 0.05 [36]. The FCGR increased by a factor of ten or more when exposed to the low-pressure hydrogen gas compared to an inert environment. Carroll and King studied four C-Mn alloys with compositions and microstructures similar to those of pipeline steels and strengths virtually equivalent to those of X42, X52 and X70 [37]. The tests were conducted in air and under low hydrogen gas pressures (101 Pa) at *R* = 0.5, *f* = 0.1 Hz, and Δ*K* values between 20 MPa m^1/2^ and 32 MPa m^1/2^. The static *K_IH_* value was found to be greater than 100 MPa m^1/2^, therefore the HA-FCG behavior was Type A (Fig. 1). The FCGR increased by about ten-fold throughout the entire range of Δ*K* values, consistent with those reported by Holbrook et al., and other studies.

### 3.2 Effect of Stress Ratio on FCG Behavior

**Fig. 6:**
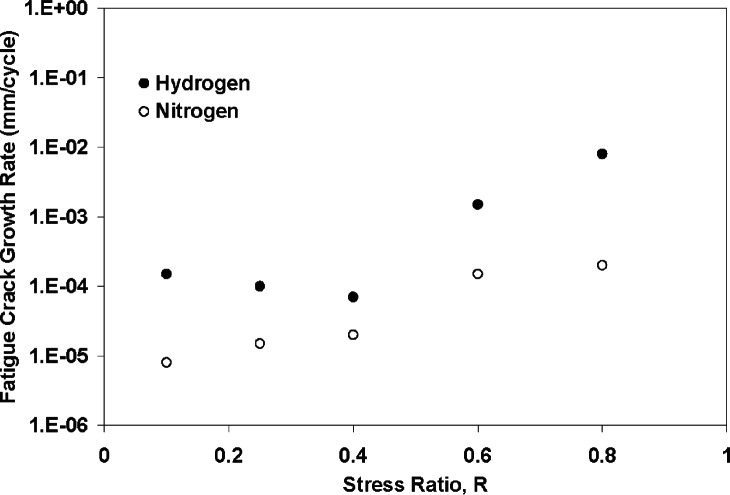
Effect of stress ratio on fatigue crack growth in X42 pipeline steel in hydrogen and nitrogen gas (6.9 MPa) (*f* = 1 Hz, Δ*K* = 10 MPa √ m) (From [6]).

Holbrook and Cialone also studied the effects of stress ratio on FCG in pressurized nitrogen and hydro gen gas [9]. Because the cyclic stress intensity range, Δ*K*, is related to *K*_max_ by the function:

(3) $$\Delta K=(1-R)K_{\max},$$

as *R* increases, the maximum applied stress intensity (*K*_max_) will be higher at a given Δ*K*. Figure 6 shows the effect of *R* on FCGR for X42 steel at a Δ*K* of 10 MPa m^1/2^. The crack growth rate in the nitrogen environment increased steadily with *R*, as would be expected, because the value of *K*_max_ would steadily increase with *R* under conditions of constant Δ*K*. Fatigue testing in hydrogen exhibited a significantly different behavior, where the FCGR’s were essentially unchanged for *R* values between 0.1 and 0.4. However, at *R* values above 0.4, the FCG’s increased at a faster rate than in nitrogen. This was attributed to the pre-mature onset of Stage III fatigue crack growth due to an HE-induced reduction in fracture toughness. Under monotonic loading conditions, the *J*-resistance fracture toughness in hydrogen was significantly less than in a nitrogen environment for the X42 alloy, and premature Stage III crack growth was attributed to a reduction in energy required for ductile crack growth. The effect of stress ratio on hydrogen-charged FCG in the X70 alloy was similar to the observations on the X42 pipeline steel. However, the fracture toughness of the X70 alloy in the inert environment appeared to be significantly lower as Stage III fatigue failure occurred at lower *K*_max_ values in nitrogen. Walter and Chandler studied the effect of stress ratio and *K*_max_ on FCG of an SA-105 Grade II steel by changing Δ*K* and *K*_min_ [38]. The tests were conducted at a frequency of 0.1 Hz and hydrogen gas pressure of ≈100 MPa. The effect of stress ratio (at constant *K*_max_) and the effect of *K*_max_ (at constant *R*) were as would be expected based on Eq. (3).

Crack “closure” or “shielding” may also influence the fatigue crack growth rate in hydrogen, compared to nitrogen, which in turn will influence the results in Fig. 6 [39, 40]. Crack closure can retard FCGR through bridging of the cracked surface around surface asperities from corrosion products, second phase particles or the crack fracture surface itself. Any study on the HA-FCG must take into account the role of crack closure, when comparing fatigue results in hydrogen with those of inert environment, or probably more significant, results from fatigue tests performed in air.

The effects of hydrogen and stress ratio on the fatigue cracking behavior of X42 steel show the intricate relationship between testing variables and environment on FCG in pipeline steels. The same material responds differently to the damage from atomic hydrogen, where at low stress ratios HA-FCG (Type A in Fig. 1) is dominant, while at high stress ratios, premature failure occurs due to HE (Type B in Fig. 1). The dramatic effects from increasing stress ratio values above 0.5 in hydrogen gas pipelines are likely in actual pipeline systems, because small fluctuations in pressure may occur frequently from compression stations or from variable wind patterns [1, 36]. On the other hand, a single pressure cycle resulting in a stress ratio of around 0.25 might occur daily as hydrogen is generated from wind and solar farms and used during peak demand. Under this type of loading cycle, Stage III fatigue crack growth may be inhibited, but at the expense of increased susceptibility to Stage II rates compared to normal rates in air, natural gas or nitrogen.

### 3.3 Effect of Gas Pressure on FCG Behavior

**Fig. 7:**
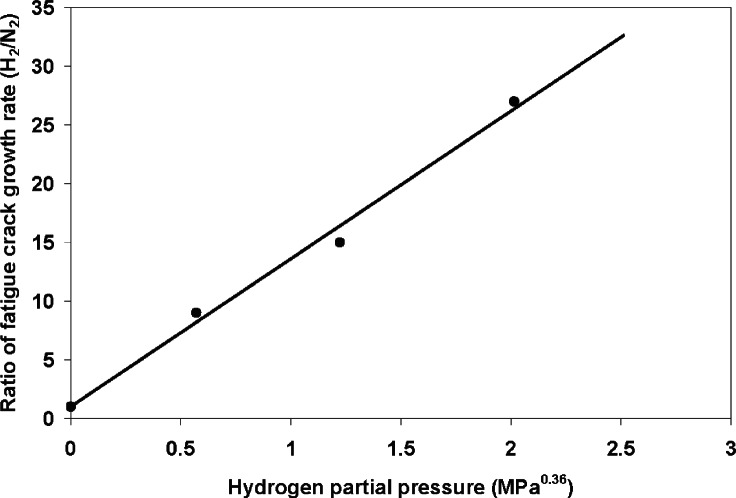
Effect of hydrogen partial pressure on the ratio ${\mathrm{da}/\mathrm{dN})}_{H_{2}}/{\left(\text{da}/\text{dN}\right)}_{N_{2}}$ for X42 steel (*R* = 0.25, *f* = 0.1, Δ*K* ≈ 22 MPa √ m). (From [8]).

Hydrogen gas pressure may also affect the fatigue behavior of a material. Holbrook et al., evaluated the effects of hydrogen gas pressure on HA-FCG in X42 steel for Δ*K* of 22 MPa m^1/2^, cyclic frequency of 0.1 Hz and stress ratio of 0.25. It was found that the ratio of FCGR in hydrogen to that in nitrogen increased as a power function (power of 0.36) of the hydrogen partial pressure, as shown in Fig. 7 for pressures up to 6.9 MPa [8]. Under equilibrium conditions, the dissolved hydrogen concentration (activity) in the steel should be proportional to the square root of the gas pressure, according to Sieverts’s law [41]. Some of this hydrogen may become irreversibly trapped, and of main concern for pressure dependent HA-FCG is the concentration of free hydrogen that can become concentrated at the fatigue crack damage zone [20]. A square root dependence is typical for the relationship between pressure and HE in static and monotonically loaded tests [42]. The lower pressure dependence in the FCG tests was attributed to nonequilibrium concentrations of hydrogen within the steel during dynamic loading, and this may be offset by performing the fatigue tests at lower frequencies.

Walter and Chandler have also studied the effect of gas pressure on SA-105 Grade II steel at pressures from 6.9 MPa to ≈100 MPa [38]. The study on pressure effects for the SA-105 steel was conducted at a stress ratio of 0.1, compared to the stress ratio of 0.25 for the plot in Fig. 7, however, the data in Fig. 6 showed that FCGR in hydrogen were not heavily influenced at these low stress ratios. The FCGR for the SA-105 steel did increase significantly when the gas pressure was raised from 6.9 MPa to 100 MPa, but there was little difference in the FCGR between 69 MPa and 100 MPa. There appears to be a pressure threshold at which the FCGR in hydrogen becomes independent of gas pressure, which is probably associated with either the maximum solubility of hydrogen in the steel or a critical hydrogen concentration in the damage zone. Further fatigue crack growth studies on pipeline steels at different pressures and frequencies should better elucidate the interplay between these two important variables and their role on crack tip hydrogen concentrations.

### 3.4 Effect of Frequency on FCG Behavior

Holbrook et al., observed no significant changes in HA-FCGR when performing tests at frequencies between 0.1 Hz and 10 Hz. This lack of dependence within this frequency range is supported by other investigators [43, 44], but a dependence on frequency has been observed by some researchers, specifically at frequencies below 0.1 Hz [38, 45]. Because hydrogen induced damage is a transport-limited phenomenon, where hydrogen must adsorb and diffuse to a highly stressed area, there is likely to be some dependence on frequency. The rate limiting steps in HA-FCG, which are controlled by cyclic frequency, are: the rate of creation of a new crack surface, the rate of hydrogen dissociation and adsorption (also dependant on the rate of crack surface repassivation), and the rate of diffusion to the crack tip plastic zone [15]. More research is needed to fully understand the rate limiting step(s) on HA-FCG in pipeline steels. This will require understanding of the surface reaction science and internal trapping and diffusion in these alloys in conjunction with fatigue testing at different frequencies (and pressures).

### 3.5 Effect of Microstructure and Yield Strength on FCG Behavior

**Fig. 8:**
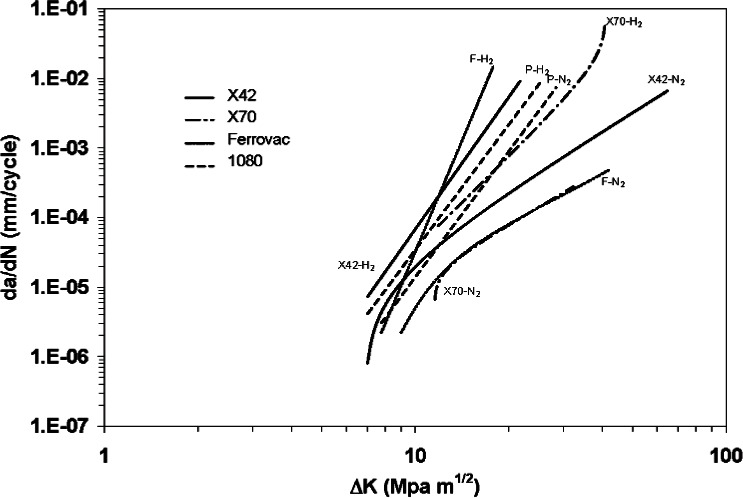
Role of ferrite and pearlite in HA-FCG (P = 1080, F = fully ferritic) (*f* = 1 Hz, *R* = 0.1, gas pressure = 6.9 MPa) (From [7, 9, 10]

Fatigue crack growth rates for steel in air or inert environments are predominantly controlled by the crack tip stress intensity, and rates are typically unaffected by microstructure [14]. However, Cialone and Holbrook have shown that this may not be the case for C-Mn steels exposed to hydrogen under certain loading conditions [7, 9, 10]. The researchers compared the FCGR for X42 pipeline steel (*σ_y_* ≈ 340 MPa), which had a ferritic-pearlitic microstructure, with the rates for a fully pearlitic (1080 steel, *σ_y_* ≈ 410 MPa) and a fully ferritic steel (*σ_y_* ≈ 110 MPa) alloy, at a frequency of 1 Hz, stress ratio of 0.1 and hydrogen gas pressure of 6.9 MPa. In addition, the results from the X70 alloy (*σ_y_* ≈ 600 MPa) can also be compared with the other steels. The complex microstructure of the thermo-mechanically rolled X70 alloy consisted of polygonal ferrite, acicular ferrite, martensite, and some retained austenite, with Nb and Mo microalloyed precipitation. Figure 8 shows the fatigue crack growth curves of the four different alloys. When the alloys were tested in nitrogen gas, the FCGR at a given Δ*K* for all of the alloys were similar, with the exception of the 1080 pearlitic steel, which showed a slight increase in FCGR at higher stress intensities. For tests conducted in hydrogen, the FCGR for all alloys were higher than those in nitrogen, especially for the fully ferritic alloy and the X42 pipeline steel. The FCGR of the fully pearlitic 1080 alloy was slightly higher in hydrogen than in nitrogen, but the increase was minimal compared to the other alloys. Unfortunately, higher FCGR for the 1080 alloy in the nitrogen environment were observed, making it difficult to make direct comparisons.

The microstructures of these steels range from 100 % ferrite to nearly 100 % pearlite (AISI 1080), with the X42 steel being intermediate, with less than 50 % pearlite in a highly banded structure oriented along the crack propagation direction. Both the fractographic observations and FCG measurements suggest that gaseous hydrogen exerts a stronger influence on ferrite than pearlite. FCG in the fully ferritic alloy occurred almost entirely along grain boundaries in hydrogen, while in the 6.9 MPa nitrogen it was essentially 100 % transgranular. Fatigue fracture through the fully pearlitic alloy in the hydrogen environment appeared to be transgranular, and light etching with 5 % nital revealed evidence of the lamellar structure that was not evident on the as-fractured surface. Cialone et al. mention that fatigue striations on the ferrite phase in the X42 steel appear to be “more widely spaced and somewhat more sharply defined” in the hydrogen environment than in the high-pressure nitrogen that was used as a control environment, but this comment cannot be easily confirmed from the fractographs presented in the paper [7]. It is noteworthy that the accelerated fatigue crack growth in a 1020 steel did not appear to have been associated with intergranular failure in the ferrite phase [36].

Other studies have examined the effects of microstructure on corrosion-fatigue and HA-FCG. Krishnamurthy et al., investigated the effect of microstructure on FCGR for API-2H steel specimens exposed to a 3.5 wt % solution of NaCl at –1.0 V (Vs SCE) using *R* of 0.1 and *f* of 0.1 Hz [46]. Surprisingly, there was little difference between FCGR for specimens with baintic, martensitic and dual phase (ferrite + martensite) microstructures at similar strength levels. Specimens of A537 steel did however show decreases in HA-FCGR as the tempering temperature was increased. Carroll and King observed no significant differences in FCGR in air or gaseous hydrogen for C-Mn pipeline type steels with different microstructures and strengths [37].

If we now compare the effects of strength level on FCG, the X70 alloy exhibited the lowest FCGR when tested in hydrogen. This contrasts with the effects of hydrogen in statically or monotonically loaded tests, where higher-strength alloys are typically more susceptible to HE. Reduction in area measurements on all four alloys were lower when tested in hydrogen, and the losses in ductility were the highest for the 1080, 100 % pearlitic alloy. The changes in tensile sample area reductions do not appear to correlate well with the FCGR results for these alloys, i.e., the ferritic alloy, which exhibited the most ductility and lowest loss in reduction in area, exhibited the highest FCGR in hydrogen. The observations of Cialone et al., with regard to the relationship between the strength of the steel and the sensitivity of fatigue crack growth to hydrogen are consistent with the results of Clark on HY-80 (*σ_y_* ≈ 650 MPa) and HY-130 (*σ_y_* ≈ 965 MPa) steels at lower hydrogen pressures [47]. Fatigue crack growth rates for the HY-80 steel ranged from 2 to 40 times those for HY-130 in 0.34 MPa hydrogen, which was in turn roughly ten times faster than for either alloy in air. In addition, Nelson found that FCGR in 1020 steel (*σ_y_* ≈ 207 MPa) at near-atmospheric hydrogen pressure increased in the low-cycle regime by more than an order of magnitude [36]. Research on modern thermo-mechanically processed, microalloyed, pipeline steels such as X100 and X120, is needed to provide further insight into the effects of strength and microstructure on HA-FCG. However, the existing literature fails to suggest a strong correlation between strength, micro-structure, and HA-FCG.

### 3.6 Effect of Inhibitor Gases

Accelerations in FCGR due to hydrogen damage may be inhibited by adding small concentrations of certain gases to hydrogen. Gases of interest would tend to adsorb on the steel surface and block hydrogen uptake onto and into the steel. Holbrook et al., [10] conducted a study on the use of inhibitor gases in hydrogen gas pipelines. X42 pipeline steel showed nearly full inhibition of HA-FCG with addition of certain gas additives such as COS, O_2_ and C_2_H_4_, where FCGR of specimens tested in the hydrogen gas mixture were similar to those tested in nitrogen. Using surface science measurement techniques such as XPS (x-ray photoelectron spectroscopy), TDS (thermal desorption spectroscopy) and AES (Auger electron spectroscopy), they found that gas additives had to meet certain criteria for beneficial inhibition. The gas additive could either block adsorption of hydrogen onto the steel surface, or it could displace hydrogen atoms that had already been adsorbed. Gases containing C, S, and O all block adsorption by forming semi-stable bonds with iron on the surface. The two gases that showed the most promise were C_2_H_4_ and O_2_; however, the addition of O_2_ to hydrogen may not be feasible because of concerns over the flammability issues and possible self-ignition associated with mixing the two gases. Nelson also showed that inhibitor gases could be used to suppress acceleration of FCGR in hydrogen [36]. In that study, CO was shown to nearly fully inhibit HA-FCG. Additions of water vapor reduced the HA-FCGR compared to rates in pure hydrogen, but not completely to the values in air. Other gases such as CH_4_, natural gas, H_2_S and CO_2_ either increased the FCGR further or had no effect on inhibiting HA-FCG.

The efficacy of gas inhibitors that adsorb on the steel surface may exhibit a change in frequency dependence compared to un-inhibited situations. Since the rate limiting step for HA-FCG may change when HA-FCG is controlled through additions of inhibitors, any future study of inhibitor gasses in HA-FCG should include evaluation of processes of loading, surface reactions with inhibitor species, and hydrogen dissociation. In addition, it is likely that trace gas impurities will exist from the production of hydrogen either from a gas well or from electrolysis, and these impurities themselves may have an effect on HA-FCG behavior. Unfortunately, these same impurities may detrimentally affect fuel cell performance, and filtering/separation processes will have to be incorporated prior to hydrogen use in the fuel cells.

### 3.7 Effect of Hydrogen on ΔK_th_

Much of the previous research on hydrogen compatibility with steel linepipes has focused on Region 2, Stage II, fatigue crack growth. However, some studies have examined the effect of hydrogen on Δ*K_th_* and the transition from Region 1 to Region 2. Testing of notched pipe sections of alloys X42 and A106B showed that the period for fatigue crack initiation was comparable and possibly even longer when testing in hydrogen [8]. The effect of hydrogen on Δ*K_th_* values for several low-alloy C-Mn steels exposed to dilute sulfuric acid under an applied cathodic potential showed that threshold stress intensity ranges may be up to 25 % lower when hydrogen charged [48]. However, the divergence between Δ*K_th_* values for charged and uncharged specimens became negligible for the lower strength steels (Vickers hardness less than 300). The fatigue crack growth thresholds of A516 steel specimens tested in high-purity pressurized hydrogen gas (*f* = 1 Hz and *R* = 0.15) were also lower than those in air [44]. Furthermore, specimens with different micro-structures exhibited different crack thresholds, with coarse-grained martensitic structures resulting in the highest Δ*K_th_*.

**Fig. 9:**
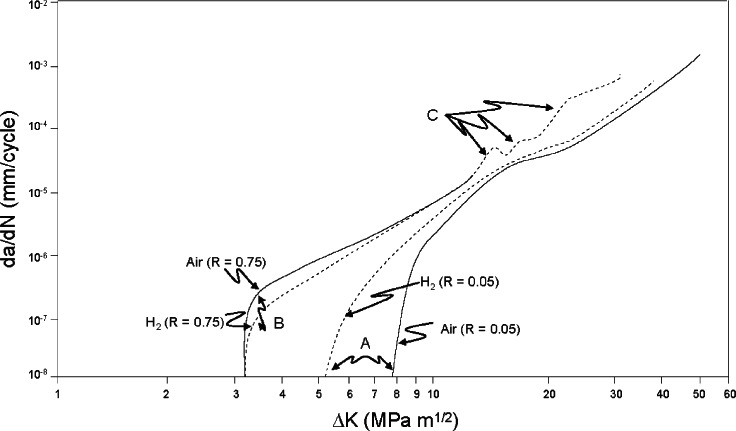
Fatigue crack growth in a bainitic 2.25 Cr-1Mo steel tested in moist air and dry hydrogen at approximately atmospheric pressure. (From [49]).

Suresh, Ritchie, and coworkers have reported an extensive body of research that, while emphasizing the effects of low-pressure (approximately atmospheric) gaseous hydrogen on near-threshold crack propagation in pressure vessel steels of the 2.25 Cr-1Mo variety, nevertheless includes results and observations at higher crack growth rates and for other varieties of steel including some pipeline steels [49]. While most of the fatigue tests wer conducted at 50 Hz, there were a limited number of tests at frequencies as low as 0.5 Hz. For the 2.25 Cr-1Mo steels, crack growth at near threshold Δ*K* values (~10^–6^ mm/cycle) is increased by low-pressure dry hydrogen compared to moist air at low stress ratios (see A in Fig. 9). The decreased Δ*K_th_* (by 30 %) in dry hydrogen and accelerated near-threshold FCGR (up to 100 %) were attributed to a lack of oxide-induced-crack closure [50]. In a separate study, the Δ*K_th_* of a similar Cr-Mo steel also exhibited lower values when tested in low pressure hydrogen gas (*f* = 10 Hz and *R* = 0.3), and the threshold value decreased further when the alloy had undergone a heat treatment that induced temper embrittlement [51]. At higher stress ratios, the threshold Δ*K* values decreased, but the effect of hydrogen was actually mildly beneficial (see B in Fig. 9) [49].

At higher crack growth rates (greater than 10^–5^ mm/s) low pressure hydrogen had little effect in 2.25 Cr-1Mo steels when the stress ratio was low. At higher *R* values (up to 0.8), significant increases (less than a factor of ten, however) were observed in the presence of low-pressure hydrogen. These increases appeared to occur at well defined values that depended on the *R* value used for the test (see C in Fig. 9).

**Fig. 10:**
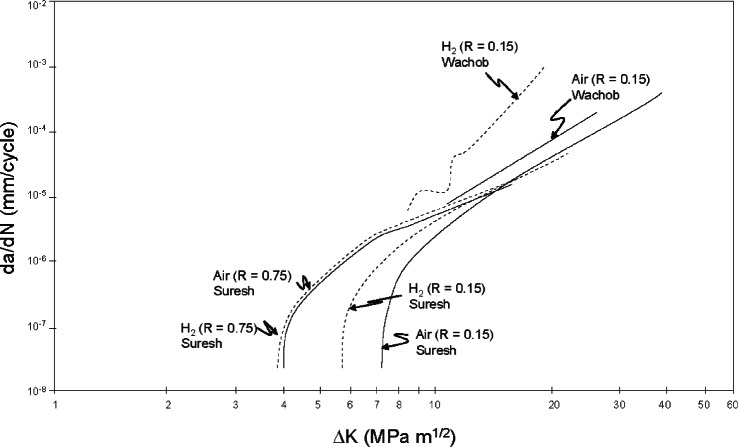
Fatigue crack propagation in X70 pipeline steel tested in air and hydrogen. Low pressure data is from Suresh et al. [49] and high pressure data is from Wachob [44].

Similar increases in near-threshold crack growth for an X70 pipeline steel in low-pressure hydrogen were also reported (Fig. 10). For X70 pipeline steel, little influence of low-pressure hydrogen was observed when FCG rates were in excess of 10^–6^ mm/cycle. Suresh et al., compare their results with those of Wachob et al., [44] for fatigue crack growth of the same steel in 6.9 MPa hydrogen gas (0.1 – 10 Hz), where significant increases in crack growth rates were observed when compared with ambient laboratory environments. Over the range of Δ*K* where the high-pressure hydrogen results of Cialone et al. [6] and Wachob et al. [44] are comparable, they are reasonably consistent.

## 4. Modeling of HA-FCG

As mentioned earlier, there are a number of mechanistic models under consideration for HE with the HELP and HEDE models currently being the most widely accepted. However, there has been far less attention toward HA-FCG. This section will focus on quantitative modeling of HA-FCG, rather than mechanistic models. Quantitative models may focus on stress and strain at the crack tip, rather than stress intensity [52]. Modeling of FCG has been done primarily from studies of enhanced crack growth in aqueous solutions, such as those by Thomas and Wei [53, 54] and Wei and Simmons [15]. Studies such as these could be put into the framework of chemical activity at the crack tip, and as such could correlate well with pressure in a hydrogen pipeline. Therefore, baseline testing of pipeline steels in pressurized hydrogen gas could then be followed by testing of cathodically-charged specimens, which are simpler and less expensive tests.

The stress intensity range, Δ*K*, is generally considered to be the primary variable controlling fatigue crack growth in both inert and hydrogen environments [15]. The magnitude of enhancement in the FCGR is proportional to approximately the square of the maximum stress intensity for constant frequency and load ratio [54], and recall that for fixed *R*, the maximum stress intensity is directly proportional to the stress intensity range (Eq. 3). Other variables that are relevant to quantitative FCGR models are; loading frequency, stress ratio, loading waveform, temperature, and stress intensity range. In order to relax codes for steel pipelines in hydrogen service, type II crack propagation as a function of loading frequency and gas pressure should be the starting point. Then, in decreasing order of importance would be stress ratio, temperature, and loading waveform.

The kinetics of HA-FCG do however depend on the stress waveform and stress ratio, at least for high-strength steels [15]. Nothing unusual is reported when the stress intensity is above the threshold for stress corrosion cracking. However, for FCG below the threshold value of stress intensity for stress corrosion cracking and for low stress ratios, hydrogen has a much smaller effect on FCG when fast-rising waveforms are used than when slow-rising waveforms (e.g., sinusoidal waveforms) are used. On the other hand, FCGR in hydrogen environments at high stress ratios is largely independent of whether fast-rising or slow-rising wave forms are used. At high stress ratios, this is to be expected, as high load ratios have relatively small changes in loading, where the limit mimics static loading.

Agreement has still not been reached on whether hydrogen diffusion or surface reactivity is the rate-limiting factor for HA-FCG in steel. Models based on either being the rate-limiting step can be found [46, 52, 53]. To date, most of these analyses have been applied to HA-FCGR data obtained in aqueous environments. Frequency effects due to the enhancement of cracking due to hydrogen may be modeled by use of exponential functions of the inverse of frequency of the form [53, 54]

(4) $${\left(\frac{\mathrm{da}}{\mathrm{dN}}\right)}_{\mathrm{enhancement}}={\left(\frac{\mathrm{da}}{\mathrm{dN}}\right)}_{\mathrm{saturation}}\;[1-\exp\left(\frac{Q}{f}\right)],$$

where *Q* is a rate constant and *f* is the loading frequency. Temperature effects are generally accounted for in the rate constant, where an Arrhenius-type expression is typically used so that an apparent activation energy can be determined for the process. However, determination of these rates for the cases of strained material near a crack tip, and the relationship between gas environment and electrochemical environments have yet to be convincingly demonstrated.

Gangloff and others have examined FCG models for corrosion-assisted fatigue cracking of steels due to hydrogen [55]. He examined the models of Wei as well as Austen’s diffusion-limited crack growth model. In addition, he examined models of both strain and stress-controlled failure. Aspects of each of these models fit the data to some extent, but none of them was definitively superior. The diffusion-limited model of Austen was nonphysical and not consistent with major aspects of the data sets. A more complete treatment of the fatigue-diffusion problem has been addressed for aluminum alloys [56].

To the author’s knowledge, modeling specific to FCG in pipeline steels under high-pressure gaseous hydrogen has not been previously reported. Work has however been reported on FCG modeling in steels, including hydrogen charging via aqueous solutions and gas charging. Use of that work, combined with FCG modeling of other materials, such as aluminum alloys, where a large body of research is available, may be applied to this case. Development of codes and standards for high-pressure hydrogen pipelines will require a systematic study that defines how much of the previous research in FCG modeling can be applied to high-pressure hydrogen gas effects on FCG in pipeline steels. Our role will be measurement of fatigue of linepipe steels in high-pressure hydrogen gas. This type of measurement is expensive to run and will not likely be required in codes, but a correlation by the electro-chemistry or corrosion communities could allow an equivalent aqueous-environment test. Modeling of the behavior would be the most useful end-product for both the code and science communities.

## 5. Summary

To summarize the available experimental observations:

1. Ferritic steels of the general type used in pipeline applications are susceptible to significantly increased fatigue crack growth rates in high-pressure hydrogen environments.
2. Unlike the situation for sustained load cracking in hydrogen environments, increases in yield strength do not necessarily bring about increased sensitivity to HA-FCG. This also appears to be the case when pipeline steels of different microstructures are considered.
3. The detrimental effect(s) of hydrogen on FCGR in these steels is dependent on hydrogen pressure.
4. Increasing the stress ratio at fixed Δ*K*, for stress ratios less than 0.5, does not significantly change the HA-FCG response. At stress ratio values above 0.5, HE mechanisms may be more prevalent as *K_max_* approaches static *K_IH_* levels.
5. In the low frequency regime (0.1–10 Hz), FCG rates in hydrogen are not particularly sensitive to loading frequency, at least for X42 type steels.
6. The work of Suresh, Ritchie and coworkers, mainly on pressure vessel type steels, indicates that the effects of hydrogen and other environments at near-threshold Δ*K* values may be very different from hydrogen effects in Region II.
7. Trace levels of certain gas species in hydrogen gas supplies may beneficially influence the FCG behavior in pipeline steels by blocking dissociation and adsorption of ionic hydrogen but may poison fuel cell performance.

## 6. Conclusions

Based on this review of the existing literature for HA-FCG in pipeline steels, fatigue will clearly be an important consideration in transportation of pressurized hydrogen gas in steel pipes. There is still much uncertainty, however, concerning the response of modern pipeline steels to HA-FCG, as well as to the variables that control HA-FCG at higher pressures and lower frequencies (below 0.1 Hz). In addition, the mechanisms and models used to predict FCGR in hydrogen may differ significantly from those used for statically loaded applications or in fatigue situations where the hydrogen derives from aqueous liquids. In order to operate safe and reliable hydrogen pipeline networks, a better understanding of the mechanisms responsible for gaseous HA-FCG is required, and more empirical data for a range of pipeline steels and test parameters must be collected.
